# Efficacy of non-obstructive aortic angioscopy for detecting a thoracic aortic graft rupture: a case report

**DOI:** 10.1186/s40792-022-01394-w

**Published:** 2022-03-14

**Authors:** Fumio Yamana, Koichi Maeda, Yuma Hamanaka, Noriko Kodani, Keitaro Domae, Masatoshi Hata, Yoshiharu Higuchi, Yukitoshi Shirakawa, Takafumi Masai

**Affiliations:** 1 Department of Cardiovascular Surgery, Osaka Police Hospital, 2 Chome-2 Yamadaoka, Suita, Osaka 565-0871 Japan; 2 Department of Cardiovascular Surgery, Osaka University Graduate School of Medicine, Osaka, Japan; 3 Department of Cardiology, Osaka Police Hospital, Osaka, Japan; 4 Department of Cardiovascular Surgery, Osaka General Medical Center, Osaka, Japan

**Keywords:** Aortic angioscopy, Vascular prosthesis graft, Non-anastomotic rupture, Contrast-enhanced computed tomography

## Abstract

**Background:**

Non-anastomotic thoracic aortic graft rupture is extremely rare and difficult to diagnose. Non-obstructive general angioscopy can help monitor the aortic intima and detect the locations of abnormal findings, while aortic angioscopy can detect vulnerable plaques in the aorta, which are difficult to visualize using conventional diagnostic methods. Herein, we report the case of a patient with non-anastomotic thoracic aortic graft rupture diagnosed using non-obstructive aortic angioscopy.

**Case presentation:**

An 85-year-old man who had undergone total arch replacement 5 years prior complained of chest pain. Emergent contrast-enhanced computed tomography (CT) revealed an intra-mediastinal hematoma around the vascular graft of the ascending aorta and angiography revealed pooling of contrast medium on the dorsal side of the vascular graft. We suspected extravasation of the thoracic vascular graft. Aortic angioscopic examination revealed a red vascular graft defect that matched extravasation at the contralateral level of the prosthetic left common carotid artery branch. Subsequently, non-anastomotic thoracic aortic graft rupture was diagnosed. The patient underwent a two-debranching thoracic endovascular aortic repair (Zone 0) with a right subclavian artery-left common carotid artery-left subclavian artery bypass. Postoperative angiography revealed disappearance of the extravasation from the graft rupture site, patent grafted vessels with flow, and no endoleak. Follow-up CT at 6 months postoperatively showed no extravasation.

**Conclusions:**

To our knowledge, this is the first report of non-anastomotic thoracic aortic graft rupture detected using non-obstructive aortic angioscopy. Aortic angioscopy can help establish a definitive diagnosis in patients with aortic graft rupture.

**Supplementary Information:**

The online version contains supplementary material available at 10.1186/s40792-022-01394-w.

## Background

Disruption of prosthetic vascular grafts can be divided into two major categories: anastomotic and non-anastomotic failures. To date, most reports have focused on anastomotic failures associated with infections [1]. There are few reports of non-anastomotic graft rupture in contemporary vascular prostheses that is caused by mechanical stress in areas, such as the groin and subclavian regions [2]. Furthermore, non-anastomotic graft rupture is extremely rare in a thoracic lesion, which makes it difficult to detect the site of disruption, especially during an emergency.

Although conventional imaging modalities, such as computed tomography (CT), magnetic resonance imaging (MRI), and transesophageal echocardiography (TEE) have been used to diagnose aortic diseases, the ability to detect millimeter-sized plaques is limited because of the relatively poor spatial resolution [3, 4]. Moreover, very few modalities can completely detect intimal injury or vulnerable plaques of the aorta in-vivo.

Herein, we report the case of a patient with non-anastomotic rupture of a prosthetic vascular graft that was diagnosed using non-obstructive aortic angioscopy which is known to help monitor the aortic intima and locations of abnormal findings.

## Case report

An 85-year-old man with a history of total aortic arch replacement performed using a 26-mm, four-branched Triplex graft (Vascutek Terumo, Tokyo, Japan) for an aortic arch aneurysm 5 years previously, was admitted to the emergency department with chest pain.

Laboratory data exhibited anemia (Hb 9.4 mg/dL), elevated inflammatory markers (WBC 8500 /μL, CRP 5.5 mg/dL), near normal renal function, elevated bilirubin level (total bilirubin 5.6 mg/dL, direct bilirubin 2.9 mg/dL), and negative blood culture results. TTE confirmed an ejection fraction of 60% and mild aortic valve regurgitation without any tamponade sign.

**Fig. 1:**
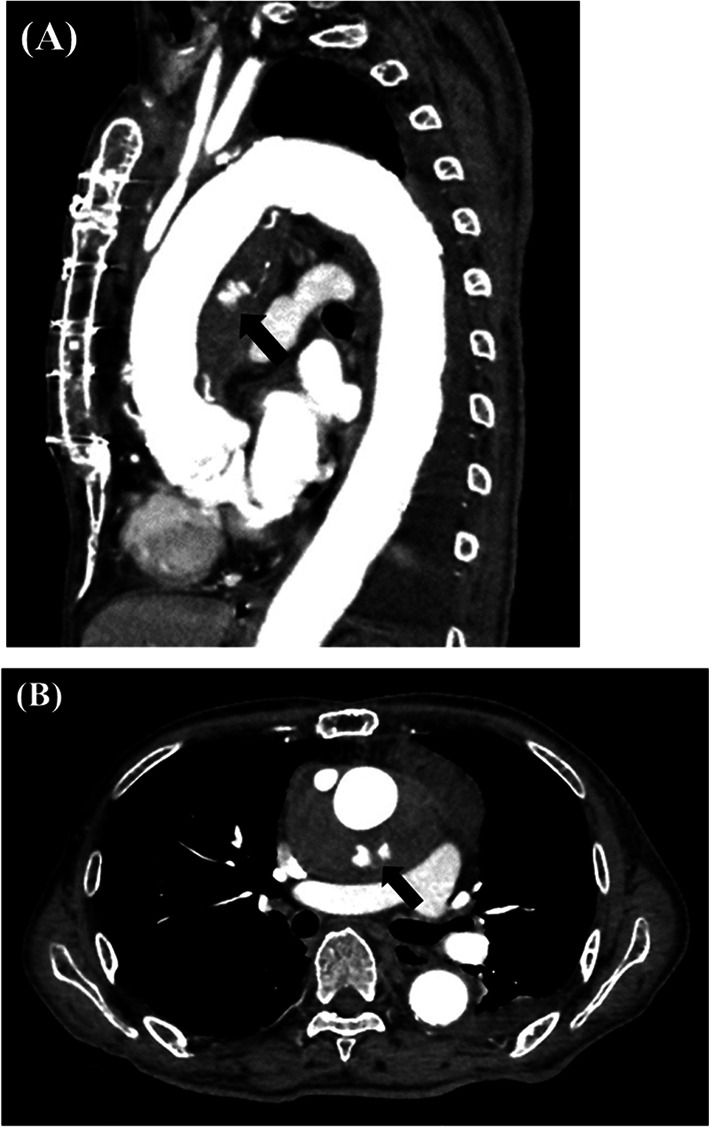
Contrast-enhanced CT findings. An intra-mediastinal hematoma around the vascular graft of the ascending aorta and extravasation on the dorsal side of the center of the vascular graft (black arrows). **A** Sagittal view; **B** Axial view

Emergent contrast-enhanced CT revealed an intra-mediastinal hematoma around the vascular graft of the ascending aorta and extravasation on the dorsal side of the center of the vascular graft (Fig. 1A, B).

**Fig. 2:**
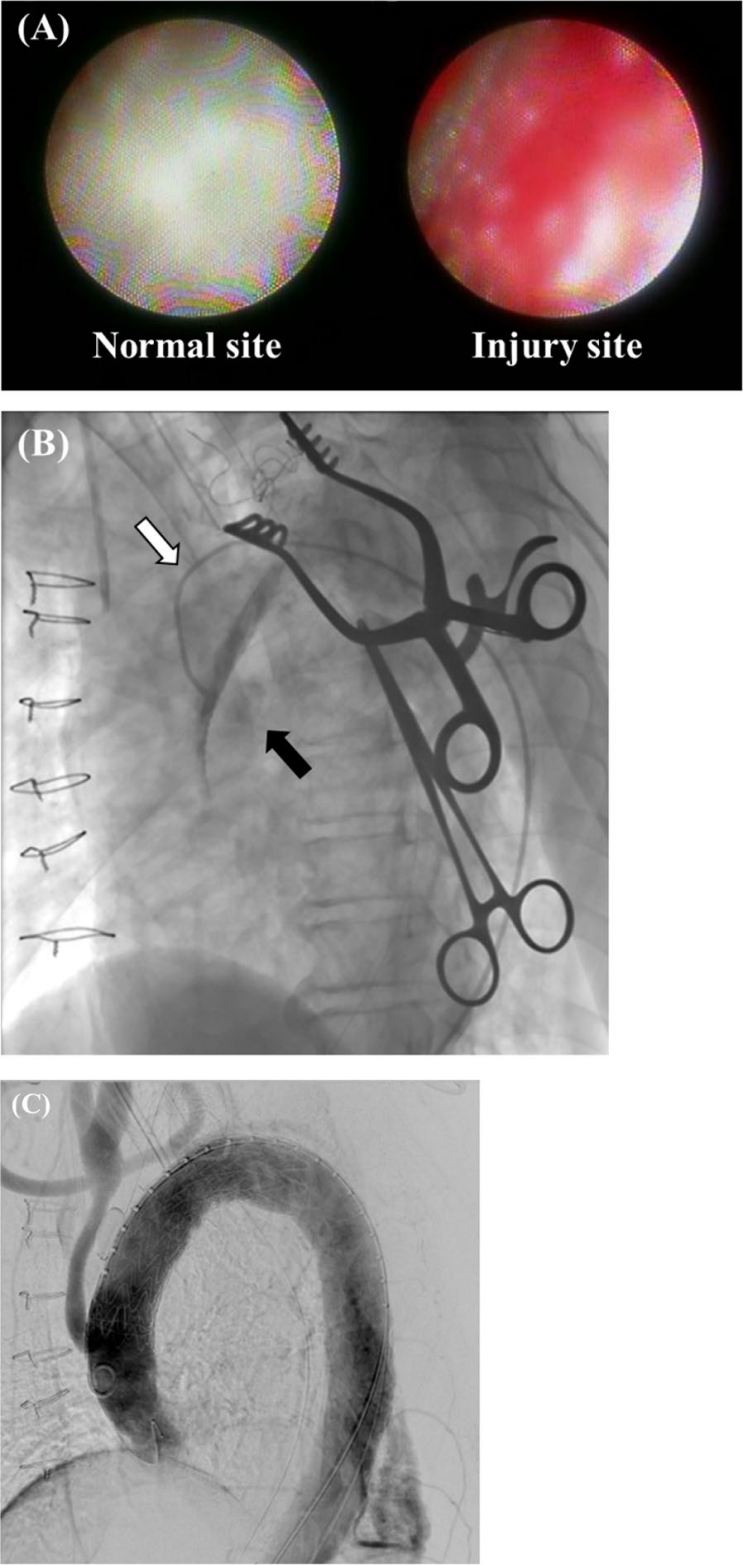
Intraoperative aortic angiography and aortoscopic findings. Aortic angioscopy shows a red graft rupture site (**A**) that matches the pooling of contrast medium (black arrow) observed on simultaneous angiography using a non-obstructive angioscopy system (white arrow) at the contralateral level of the left common carotid artery branch contralateral level (**B**). Post-deployment angiography shows no extravasation (**C**)

Emergent angiography, which was performed under general anesthesia in a hybrid operating room, revealed pooling of contrast medium on the dorsal side of the vascular graft. However, the source of the leak was unclear. Aortic angioscopic examination of the graft was performed using a non-obstructive angioscopy system (FT-203F angioscope and VISIBLE fiber; Fiber Tech Co., Ltd., Tokyo, Japan). This revealed a red vascular graft defect (Additional file 1: Video S1, Fig. 2A) that matched the simultaneous angiographic extravasation at the contralateral level of the prosthetic left common carotid artery (CCA) branch (Fig. 2B). A distance of 2 cm between the first branch of the graft vessel and rupture site was confirmed using a marker catheter.

We confirmed non-anastomotic graft rupture and immediately started two-debranching thoracic endovascular aortic repair. The left CCA and left subclavian artery (SCA) were revascularized via right SCA-left CCA-left SCA bypass using an 8-mm T-shaped ring-enforced polytetrafluoroethylene graft. A cTAG stent-graft (34 mm × 150 mm; W.L. Gore and Associates, Flagstaff, AZ) was deployed just distal to the first graft branch of the brachiocephalic artery via a femoral approach.

**Fig. 3:**
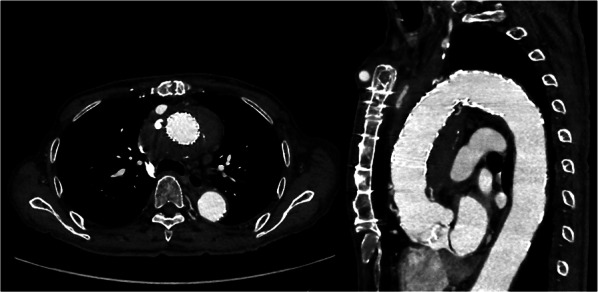
Postoperative computed tomography findings. Postoperative computed tomography shows no extravasation

Finally, embolization at the left SCA was performed using the Vascular Plug (12 mm), and the proximal left CCA of the bypass anastomosis was closed with a Hem-o-lok XL (Research Triangle Park, NC, USA). A final angiography revealed the disappearance of the extravasation from the graft rupture site, patent flow to the grafted vessels, and no endoleak (Fig. 2C). The postoperative course was uneventful. He was transferred for rehabilitation in postoperative day 13. Follow-up CT at 6 months postoperatively showed no extravasation (Fig. 3).

## Discussion

Rupture after thoracic aortic surgery is usually caused by anastomotic failure. The literature reports a frequency of 1–12%, and the diagnosis is usually made 8–10 years after the original surgery [5]. As a differential diagnosis, rupture of an artificial vascular graft may occur although it has not been reported in the thoracic region. The most reported cause of graft rupture is infection. Moreover, there are reports of physical damages caused by aging, suturing during a previous surgery, calcification of the wrapped aorta, and contact with the ribs [6–9].

In this case report, blood tests at the time of admission showed elevated inflammatory markers but negative blood culture results. Therefore, infection was ruled out as a cause for the graft rupture.

In addition, the rupture site of the vascular prosthetic graft was on the dorsal side of the prosthetic graft, and aortic wrapping was not performed in the previous surgery. Therefore, it is unlikely that the graft rupture was caused by physical damage, and the cause of graft rupture was unknown.

Furthermore, a rupture site is sometimes difficult to detect despite using CT or angiography especially in the emergent case. When the rupture site is a proximal anastomosis, open surgery is the only treatment option. However, it is possible to perform endovascular treatment for a rupture of the graft itself or the distal anastomosis site. Therefore, detection of the rupture site was crucial in our case, because the patient had a high risk of open repair due to age and frailty.

Graft rupture was suspected on imaging. However, since graft rupture is extremely rare, we had to make a definitive diagnosis. Therefore, we prepared to treat the patient as soon as the rupture site was confirmed and performed angioscopy in the hybrid operating room. Angioscopy helped determine the rupture site and we immediately started two-debranching thoracic endovascular aortic repair.

Non-obstructive general angioscopy is a novel method that may have important implications for the diagnosis and management of vulnerable atherosclerotic plaque [10, 11]. Furthermore, this modality can help monitor the aortic intima and locations of abnormal findings in patients with a shaggy aorta and chronic type B aortic dissection [12], which are difficult to visualize using conventional diagnostic methods. Therefore, angioscopy has been used in endovascular therapy to confirm the location of arteriosclerotic plaques to prevent embolisms during aneurysm treatment or confirm the location of primary entry and re-entry in cases of aortic dissection [10, 12]. In the current case, aortic angioscopic examination of the graft was performed using a non-obstructive angioscopy system, as previously reported [10]. This revealed the rupture site to be a red thrombus on the vascular graft, while the graft wall around the distal anastomosis to the rupture site was intact. These findings have not been previously reported.

## Conclusion

We reported the efficacy of non-obstructive aortic angioscopy for definitive diagnosis in a patient with graft rupture.

## Supplementary Information

**Additional file 1: Video S1.**

## Abbreviations

CT: Computed tomography
MRI: Magnetic resonance imaging
TEE: Transesophageal echocardiography
LCCA: Left common carotid artery
LSCA: Left subclavian artery
